# Laparoscopic image of carbon dioxide embolism during laparoscopic hepatectomy: a case report

**DOI:** 10.1186/s40981-020-00345-4

**Published:** 2020-05-30

**Authors:** Kenichi Takechi, Mari Ito, Yi Peng, Waka Daizen, Ichiro Shimizu

**Affiliations:** Matsuyama Red Cross Hospital, 1 Bunkyocho, Matsuyama City, Ehime Japan

**Keywords:** Pulmonary embolism, Laparoscopy complications, Pringle maneuver

## Abstract

**Background:**

Carbon dioxide embolism is a life-threatening complication of laparoscopic hepatectomy.

**Case presentation:**

A 59-year-old man was admitted for laparoscopic hepatectomy. Approximately 5 h after commencing the operation, we observed a gradual decline in the SpO_2_ from 100 to 94%, reduction in the ETCO_2_ from 44 to 19 mmHg, reduction in the systolic blood pressure from 100 to 82 mmHg, and elevation of the heart rate from 82 to 120 beats/min. Intraoperatively, the image displayed on the laparoscopic monitor revealed a small tear in the vein. The inspired O_2_ fraction was raised to 1.0, intravenous phenylephrine (0.1 mg bolus) was administered, and the respiratory rate was increased. After the patient was stabilized, the injured vein was cut and sealed. After the embolic event, the entire operation was completed without complications.

**Conclusions:**

Careful observation of the laparoscopic monitor is important, particularly during establishment of pneumoperitoneum in patients undergoing laparoscopic hepatectomy.

## Background

Compared with open hepatectomy, laparoscopic hepatectomy is a minimally invasive procedure associated with lesser intraoperative bleeding and postoperative pain and faster recovery. Carbon dioxide (CO_2_) is commonly used for pneumoperitoneum during laparoscopic procedures, and CO_2_ embolism is an uncommon but life-threatening complication of laparoscopic hepatectomy [1]. Low central venous pressure (< 5 mmHg), high-pressure pneumoperitoneum (> 12 mmHg), and the Pringle maneuver are used to achieve hemostasis during laparoscopic hepatectomy [2]. However, intra-abdominal insufflation pressures exceeding the central venous pressure theoretically predispose patients to CO_2_ embolism, particularly when performing operations involving highly vascular solid organs such as the liver. Reportedly, the incidence of CO_2_ embolism during laparoscopic hepatectomy (1.2–4.6%) was approximately 10-fold higher than the overall incidence of CO_2_ embolism during laparoscopic surgery (0.15%) [1, 3, 4]. Usually, dissociation between the end-tidal carbon dioxide (ETCO_2_) and arterial gas CO_2_ is used to diagnose CO_2_ embolism. We describe CO_2_ embolism that gas entry was confirmed on a laparoscopic monitor in a patient who underwent laparoscopic hepatectomy using the Pringle maneuver.

## Case presentation

A 59-year-old man (height 171 cm, weight 74 kg) was admitted to our hospital for laparoscopic right hemihepatectomy for a diagnosis of intrahepatic cholangiocarcinoma. The operation was performed under combined general and epidural anesthesia. Preoperative laboratory findings were within normal limits, and no premedication was administered to the patient. A standard anesthetic protocol was implemented, including routine non-invasive arterial blood pressure monitoring, electrocardiography, and oxygen saturation (SpO_2_) measurement on arrival at the operating room, and his vital signs were stable. Before induction of general anesthesia, a thoracic epidural catheter was inserted into the thoracic 7–8 interspace. Anesthesia was induced using propofol (2 mg/kg), remifentanil (0.3 μg/kg/min), and rocuronium (0.8 mg/kg) and was maintained using desflurane (4–6%), remifentanil (0.15–0.3 μg/kg/min), and rocuronium (5 μg/kg/min). The following ventilator settings were used: tidal volume 7 mL/kg ideal body weight, inspiratory:expiratory ratio 1:2, inspired O_2_ fraction 0.6 with air, and inspiratory fresh gas flow 2 L/min. The respiratory rate was adjusted to 8–16 breaths/min to maintain an ETCO_2_ pressure of 30–45 mmHg. After induction of anesthesia and tracheal intubation, a 22-G catheter was inserted into the left radial artery for blood sampling and continuous blood pressure monitoring. A central venous catheter was inserted from the right jugular vein for continuous central venous pressure monitoring. The patient was placed in the reverse Trendelenburg position with a left tilt, and CO_2_ was insufflated to create pneumoperitoneum (intra-abdominal pressure 12 mmHg). A low central venous pressure (< 5 mmHg) was maintained throughout the operation.

**Fig. 1:**
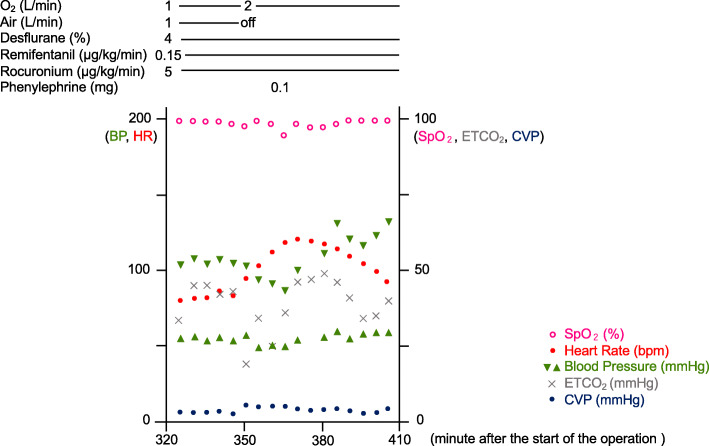
Vital signs during the CO_2_ embolism

**Fig. 2:**
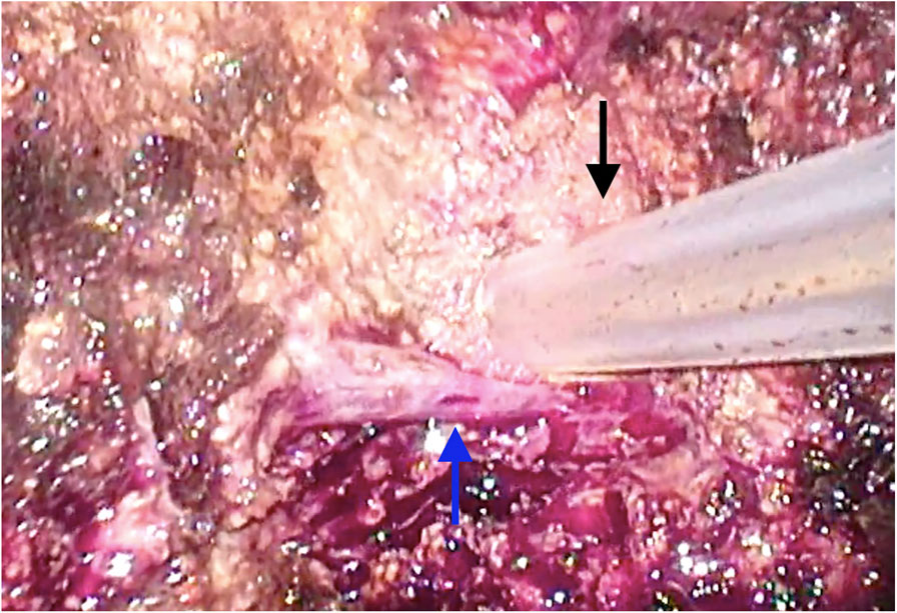
Laparoscopic image of the surgical field. A small laceration without bleeding is detectable in the wall of a vein on the surface of the resected right hepatic lobe (blue arrow) during surgery using an ultrasonic surgical aspirator (black arrow)

Approximately 5 h after commencing the operation and establishment of pneumoperitoneum, during the Pringle maneuver (hepatic resection performed with clamping the branches of the vascular pedicle), we observed a gradual decline in the patient’s SpO_2_ from 100 to 94%, reduction in the ETCO_2_ from 44 to 19 mmHg, reduction in the systolic blood pressure from 100 to 82 mmHg, and rapid elevation of the heart rate from 82 to 120 beats/min (Fig. 1). Intraoperatively, the image displayed on the laparoscopic monitor revealed a small tear in the wall of a vein without bleeding but ballooning with gas within the vessel (Fig. 2, Supplementary video 1). CO_2_ embolism was suspected, and arterial blood gas testing was performed for confirmation (pH 7.153, partial pressure of O_2_ 122.8 mmHg, partial pressure of CO_2_ 78.2 mmHg). The pneumoperitoneum pressure was reduced to 10 mmHg, and the Pringle maneuver was abandoned. The inspired O_2_ fraction was immediately raised to 1.0, and intravenous phenylephrine (0.1 mg bolus) was administered to treat hypotension. The respiratory rate was increased to wash out CO_2_. After the patient was hemodynamically stabilized, the injured vein was immediately cut and sealed. Approximately 4 h after this embolic event, the entire operation was completed without complications. Intraoperative blood loss was 550 ml, and the patient was safely extubated. His postoperative course was uneventful, and he was discharged without further complications.

## Discussion

In this case, CO_2_ embolism occurred during establishment of pneumoperitoneum in a patient undergoing the Pringle maneuver. Optimal hemostasis is extremely important during laparoscopic hepatectomy [5]; notably, specific hemostatic modalities are being developed for laparoscopic hepatectomy. High-pressure pneumoperitoneum (10–14 mmHg), low central venous pressure (< 5 mmHg), a carefully controlled pneumoperitoneum gas delivery system, and the use of appropriate hemostatic devices are recommended for safe laparoscopic hepatectomy with minimal bleeding [2]. However, low central venous pressure with high-pressure pneumoperitoneum results in a pressure gradient that predisposes patients to CO_2_ embolism. Clamping the hepatoduodenal ligament interrupts blood flow through the hepatic artery and the portal vein and controls bleeding from the liver. This hemostatic technique, referred to as the Pringle maneuver is commonly used during laparoscopic hepatectomy. During the Pringle maneuver, bleeding occurs secondary to backflow from the hepatic veins, and intrahepatic vascular pressure is usually the same as the central venous pressure. Makabe et al. reported that hepatic artery occlusion increases the risk of CO_2_ embolism during laparoscopic hepatectomy in a pig model [6]. The low central venous pressure, high-pressure pneumoperitoneum, and the Pringle maneuver all contributed to CO_2_ embolism in our patient. Clinicians should be mindful of the risk of CO_2_ embolism, particularly during establishment of pneumoperitoneum in patients undergoing surgical procedures that involve the Pringle maneuver.

CO_2_ embolism typically manifests with a sudden decrease in ETCO_2_, reduction in blood pressure, and elevated heart rate. Transesophageal echocardiography (TEE) is one of the most sensitive and specific diagnostic modalities for intraoperative CO_2_ embolism [7]. However, TEE is not routinely used for laparoscopic hepatectomy owing to its invasiveness. Therefore, intraoperative CO_2_ embolism is usually diagnosed based on clinical symptoms. Using a high-resolution laparoscopic camera, even minute structures in the operative field could be projected as a wide and clear image on a high-resolution monitor in our case. Therefore, the surgeons and anesthesiologist could immediately detect the small tear in the wall of the injured vein. These images facilitated prompt diagnosis of CO_2_ embolism by the surgical team.

Patients with suspected CO_2_ embolism should be ventilated using inspired O_2_ fraction 1.0 to absorb the bubbles and combat the ventilation mismatch to improve hypoxemia [8]. The pneumoperitoneum pressure should be reduced or conversion to open surgery is warranted to release intra-abdominal gas and prevent further embolism [8]. In this case, the surgical team concurred that abandoning the pneumoperitoneum could increase the risk of bleeding from the vein with consequent difficulty in treating the injured vein. Releasing the Pringle maneuver could theoretically increase hepatic blood flow and reduce the pneumoperitoneum pressure-venous pressure gradient. Therefore, we reduced the pneumoperitoneum pressure, abandoned the Pringle maneuver, and continued the laparoscopic procedure. Another possible strategy to prevent progressive CO_2_ embolism is patient positioning (the Trendelenburg position with a left tilt) and high positive end-expiratory pressure (PEEP). ventilation [9]. However, the Trendelenburg position may interfere with optimal visualization of the operative field during laparoscopic hepatectomy, and high PEEP ventilation may increase the right atrial pressure with a risk of paradoxical embolism through a latent patent foramen ovale [10].

## Conclusion

The Pringle maneuver performed during laparoscopic hepatectomy may theoretically predispose patients to CO_2_ embolism. Laparoscopic imaging provides important information to enable prompt diagnosis and treatment of CO_2_ embolism by facilitating effective communication between the surgeon and anesthesiologist. Careful observation of the laparoscopic monitor and rapid communication between the surgeon and the anesthesiologist are important, particularly during the establishment of pneumoperitoneum in patients undergoing operations that involve the Pringle maneuver.

## Supplementary information

**Additional file 1:.** Supplementary video

## Data Availability

Not applicable

## Abbreviations

CO_2_: Carbon dioxide
ETCO_2_: End-tidal carbon dioxide
SpO_2_: Oxygen saturation
PEEP: Positive end-expiratory pressure
TEE: Transesophageal echocardiography
